# A twofold tale of one mind: revisiting REC’s multi-storey story

**DOI:** 10.1007/s11229-020-02857-z

**Published:** 2020-09-05

**Authors:** Erik Myin, Jasper C. van den Herik

**Affiliations:** 1 Centre for Philosophical Psychology, Department of Philosophy, University of Antwerp, Prinsstraat 13, 2000 Antwerpen, Belgium; 2 Department of Psychiatry, Amsterdam UMC, Meibergdreef 9, 1105 AZ Amsterdam, The Netherlands

**Keywords:** Radical enactivism, Skill, Content, Reflexivity

## Abstract

The Radical Enactive/Embodied view of Cognition, or REC, claims that all cognition is a matter of skilled performance. Yet REC also makes a distinction between basic and content-involving cognition, arguing that the development of basic to content-involving cognition involves a kink. It might seem that this distinction leads to problematic gaps in REC’s story. We address two such alleged gaps in this paper. First, we identify and reply to the concern that REC leads to an “interface problem”, according to which REC has to account for the interaction of two minds co-present in the same cognitive activity. We emphasise how REC’s view of content-involving cognition in terms of activities that require particular sociocultural practices can resolve these interface concerns. The second potential problematic gap is that REC creates an unjustified difference in kind between animal and human cognition. In response, we clarify and further explicate REC’s notion of content, and argue that this notion allows REC to justifiably mark the distinction between basic and content-involving cognition as a difference in kind. We conclude by pointing out in what sense basic and content-involving cognitive activities are the same, yet different. They are the same because they are all forms of skilled performance, yet different as some forms of skilled performance are genuinely different from other forms.

## Mind the gaps

According to the Radical Enactive/Embodied view of Cognition, REC for short, all cognition is a matter of organism/environment interaction that is best explained in terms of learning, adaptation, and change acquired through a history of preceding interactions (see Hutto and Myin 2013, p. 8). REC insists that cognitive activities that are shaped as a consequence of such interactions need not be contentful in themselves nor need they be explained in terms of inner or mental content-carrying representations. In fact, REC has argued extensively that cognitive activity can be flexible and adaptive to a broad range of, sometimes unpredictable, circumstances, without this involving contentful information processing of any kind (Hutto and Myin 2017, pp. 118–119, pp. 140–143).

REC thereby rejects a standard motivation for introducing contentful information processing as a necessary ingredient for all kinds of cognition: the flexibility that contentful representation allegedly brings to the table. According to a standard line of content-friendly reasoning, only by adding contents to the mix can responses to the environment become anything more than rigid and blindly repetitive behaviours. This is because cognition only arises, so it is claimed, via the contribution of contentful mental information processing, which covertly guides overt behaviour (Adams and Aizawa 2008; Miłkowski 2013 Ch. 1; O’Brien and Opie 2004). Building on empirical work concerning motor activity, and resonating with a converging line of critique of cognitivism based on the “frame-problem”, REC inverses that reasoning: given the number of contextual variables actually adapted to in actual organismic reactions, any system that is based on activity-guiding contents is bound to become inflexible and unprepared for any environmental contingency that has not-yet been contentfully specified (Myin and Hutto 2013, Ch. 3, see also Keijzer 2001; Flament-Fultot 2016, 2019). The REC-friendly alternative holds on to learning, adaptivity and plasticity while simultaneously letting go of the idea that contentful representations guide activity (Hutto and Myin 2017, Epilogue; Flament-Fultot 2016, 2019). REC thus endorses Jerry Fodor’s worst nightmare, i.e., that “abilities are prior to theories”, that “competence is prior to content” and that “knowing how is the paradigm cognitive state and (…) is prior to knowing that” (Fodor 2008, p. 10).

Yet REC also acknowledges that content-involving cognition exists. Some cognitive activities, such as making a judgment, consist in ways of talking or thinking that can be evaluated for truth and falsity and therefore involve content. REC thus rejects really Radical Enactive/Embodied Cognition, according to which cognition never is content-involving (Van den Herik 2014; Harvey 2015; Rosenberg 2015). Moreover, REC has proposed that cognitive capacities that occur in basic forms can become content-involving in different contexts (Hutto and Myin 2017; Myin 2020). For example, basic episodes of remembering an object or another organism by re-enactive re-experiencing can evolve into autobiographical memory in subjects that have acquired narrative capacities. That is, though a re-enacted experience does not by itself tell at which time it happened, a person capable of narrating this experience is able to situate such an experience at a specific location in her personal history (Hutto and Myin 2017, chapter 9). She can come to recollective judgments, such as “I wore that green dress on my 18th birthday”. REC’s story about content-involving cognition is therefore a two storey-story: one containing both contentless and content-involving threads. According to REC, the story of content-involving cognition is told in terms of certain sociocultural practices that constitute the context in which content becomes possible. In particular, once truth-telling practices are in place, certain cognitive activities become evaluable for truth and falsity and thereby become content-involving. Under these conditions it becomes possible to construct an ideal of truth as a standard beyond ourselves, which we bind to, and to formulate ‘the pursuit of truth” as a goal in itself (Price 2013; Hutto and Myin 2017, pp. 119–120).

REC also claims that cognitive possibilities before and after truth-telling practices arrived on the scene are significantly different and constitute a difference in kind. Due to the nature of the difference involved, REC has described the development from basic to content-involving cognition as kinky. A kinky line contains no gap. But it does contain a change that implies a genuine difference before and after that change.

REC’s multi-storey story might seem to lead to problematic gaps. First, it can be thought that, by making a fundamental difference between basic minds and content-involving minds, REC introduces an *interface problem* between these two minds. REC would thus be unable to account for how these two minds come together in contexts in which content is involved. Here REC’s two storey-story is taken to be a story of two minds. And these two minds, so the concern goes, act like water and oil: they don’t mix even in circumstances when they are brought closely together. According to this objection, REC’s claim that basic minds become content-involving by becoming embedded in content-involving practices is empty because REC has no account of how such embedding is possible.

There is another alleged problematic gap. By its insistence on a ‘kinky’ ‘categorical difference’ between contentless and content-involving cognition, REC makes a principled distinction between animal and human cognition. Such a contrast, so this second concern goes, is not compatible with the recognised continuity between humans and animals. Indeed, it is claimed that REC creates a chasm where there is only gradual difference and so denies the animal nature of our own minds.

In this paper, we revisit REC and its two storey-story in the light of these concerns; we show the way REC’s two-storey story can and should be understood so that it does not give rise to either the interface problem or a problematic gap between humans and animals. In short, we insist that the two storeys belong to the story, not to the mind. For there is and remains only one mind, even when that mind becomes content-involving. All forms of cognition, irrespective of whether they are basic or content-involving, are a matter of the exercise of abilities, competence and know-how. At the same time, there are different kinds of abilities, competences and know-how. Though swimming and playing chess are both manifestations of abilities, only in playing chess can one checkmate an opponent. What makes the move of a chess piece a checkmate is that it is a move in a particular normative practice that has developed out of other practices and activities. And so it is with content-involving abilities: they have a special character but that special character is due to particular practices that have emerged in the natural history of the human species (Hutto and Satne 2015).

With both the underlying continuity and the role of context in place, notably the context of sociocultural practices, REC’s answer to the two alleged gaps mentioned above can take shape. In this response, the view of the mind that fuels the interface problem is eschewed: a picture of the mind as consisting of two components, one contentless and one content-involving, which need to “interface” every time a content-involving activity is engaged in is rejected. Moreover, contents are not carried by inner entities. Nevertheless, some activities, are, in the right circumstances, contentful. Particular circumstances allow for the kinds of competence and cognition which, despite not requiring underlying special or unique ingredients, are novel and different with respect to what came before. Human and animal minds then are the same but different. They are the same in that they are built of similar ingredients. It is not the case that human minds contain some extra-ordinary cognitive mechanism or a special architecture lacking in non-human minds. Nor are the processes that lead to content-involving practices unique, in the sense that they contain evolutionary operations or principles absent in evolution in general. But similar ingredients do not necessarily lead to similar outcomes. For example, it is possible to use stone in different settings via different chains of similar activities to build different buildings—think about a stone house and a medieval military castle or a gothic cathedral. And once one has a castle, or a cathedral, further activities then become available, which are out of reach to the inhabitant of the humble house. Hence, a continuous process can lead to an outcome that is categorically different from its initial stage. That is why it is possible to claim that the emergence of content is kinky without this flouting a principle of evolutionary continuity.

Here is how we proceed. To prepare the ground, in Sect. 2, we first revisit REC’s two-pronged story about cognition. This allows us to clarify the notion of content. Then we deal with the alleged gaps. Sect. 3 is devoted to the concern that REC entails an interface problem. We argue that this problem only arises if assumptions are made about what makes cognition content-involving that are contrary to REC. In Sect. 4, we turn to the concern that REC entails a problematic gap between humans and animals. We argue that REC is entitled to recognise a fundamental difference between basic and content-involving cognition. In the conclusion, we detail how basic and content-involving forms of cognition can be understood as being the same but different in a way that avoids contradiction or paradox.

## The two storey-story

It is widely assumed in the still influential cognitivist outlook on the mind that contents are everywhere in cognition. This is because it is assumed that the main phenomena to be explained, the crucial cognitive *explananda*, in perception, motor control, memory and thought, are contentful states, or states carrying representational content. Let’s call the claim that the main cognitive phenomena are contentful, the P-claim. But content cuts even deeper on the standard representational views. For the main *explanantia* for cognition are also taken to be contentful. That is, in order to explain the principal cognitive states, reference needs to be made to other contentful states that operate in producing or changing those cognitive states. Let’s call this representationalist explanatory assumption the E-claim.

Enter REC. REC rejects both the P and the E-claim in their unqualified form. For REC, it is not the case that *all* cognitive phenomena are content-involving nor is it the case that *all* explanantia are content-based. Instead, REC holds that only *some* cognitive explananda are content-involving, and that in *some* cases these can be explained in terms of other content-involving activities.

REC holds that any advance in the debate on representational content requires a well-defined and substantive understanding of what content is. Such an understanding is available in the commonly endorsed definition of content, namely content is what characterises or specifies something else, in a certain way, such that this characterization or specification is evaluable in in terms of truth or accuracy (Travis 2004, p. 59; Crane 2009). Uttering a declarative sentence, such as “Snow is white” can serve as a paradigm for a content-involving activity. It specifies something, namely the colour of snow, in such a way (with a description in the English language) that it is evaluable for truth or falsity.

REC accepts this substantive notion of content. Still, REC rejects the claim that all cognitive explananda are content-involving. According to REC, many cognitive phenomena that are standardly taken to be content-involving are not in fact content-involving at all, such as instances of perception, imagination or remembering. For example, unlike what is commonly assumed, perception is not “like the newspaper”, informing its intended readership by delivering content-carrying descriptions (see Siegel 2016 for such a picture of perception). Rather, basic perception is “silent” (Travis 2004, 2014). Contentless cognition is, according to REC, phylogenetically and ontogenetically basic.

Since REC does not deny the existence of content-involving cognition, it also does not reject the P-claim for all of cognition. REC recognises that some instances of cognition, such as asserting “Snow is white”, are indeed content-involving. Content arises, so REC claims, only when socially interacting individuals with basic abilities come to engage in certain communicative practices, in particular truth-telling practices. In these practices, it becomes possible to engage in activities, such as making an assertion, that are evaluable for truth or falsity (Hutto and Myin 2017, pp. 90–91).

Content-involving practices build on contentless capacities. As detailed in Hutto and Myin (2017), perception, imagination and memory can become content-involving once the proper circumstances are in place. Take memory. According to REC, a basic episode of remembering can consist of a re-enactment of a previous experience. For example, an animal, upon casting its gaze on the tree it is approaching, can re-enact what happened before: it experienced a dog storming menacingly forward from behind it. Such a re-enactment can consist of nothing more than the partial re-experiencing of what happened before. In that sense it would be imagistic, akin to seeing a scene and contentless. It does not specify when the original experienced event happened, where it occurred, or any other description. In other words, a re-enactment does not necessarily involve the claim that the re-enacted event unfolded in a particular way. This follows if perception is basic. This account avoids having to make assumptions that create deep explanatory puzzles, such as that there is ‘[…] some feature of our memory image that tells us the time to which it belongs’ (Wittgenstein 1992, p. 5).^1^

Therefore, such basic re-enactments are a far cry from being episodes of autobiographical remembering. On a standard construal of autobiographical memory, this is understood as:

that uniquely human form of memory that moves beyond recall of experienced events to integrate perspective, interpretation, and evaluation across self, other, and time to create a personal history. To put it succinctly, autobiographical memory is memory of the self interacting with others in the service of both short-term and long-term goals that define our being and our purpose in the world (Fivush 2011, p. 559).

REC argues that basic remembering can evolve into fully-fledged autobiographical remembering. For this to happen, there needs to be a context in which rememberers have acquired content-involving capacities for speaking, judging and narrating. In such a context, narrative capacities can be brought to bear on remembering. Re-enacting can thereby become interlaced with narrating. It thus becomes possible to remember autobiographically (see Hutto and Myin 2017, chapter 9). Now one can remember events *as* having occurred in one’s personal past: one can recall the event featuring the neighbour’s dog, *as* that dog storming forward towards oneself last Friday, during a walk round town after dinner. And now one can make memory-based claims which are content-involving (“Surely, I experienced that person’s dog behaviour last Friday as threatening!”), or ask questions about the episode which are evaluable for truth or falsity (“Was it really the neighbour’s dog that stormed forward?”).^2^

So REC does not reject the P-claim for all of cognition. It accepts that some cognitive phenomena are content-involving. But what about the E-claim, that is, what about the explanantia for cognition? REC allows that content-involving activities can play the role of explanantia, as when one explains what someone does according to a previously entertained plan or when one explains how a learner acts by being verbally instructed by a teacher. REC thus happily accepts content-involving explanantia, in so far as these explanantia are themselves explained in REC-friendly ways. However, as REC holds that there is no such explanation for *mental* contentful representations (Hutto and Myin 2013), it does not accept them as explanantia. This rejection holds across the board: reference to contentful mental representations is not needed to explain either basic or content-involving cognition (Hutto and Myin 2014).

Concerns can and have been voiced over this story. A prominent concern is that it contains problematic gaps. The interface between basic and content-involving minds is one such gap, to which we now turn.

## Being in two minds

Worries about REC’s creation of an interface problem target the relation between basic and content-involving cognition. Roughly, the concern is that once a fundamental difference is made between basic and content-involving minds, it becomes impossible to account for how they come together in concrete instances. Martin Weichold expresses such reasoning when he writes about REC:

“For instance, how should one think about the interaction, or the interface, of basic and nonbasic minds? For example, think of a person walking to a theater: Her walking might be analyzed as dynamical interaction of a basic mind with worldly offerings of its environment. But likewise, the person is straightforwardly walking toward the theatre: the behavior is also guided by a contentful intention in action. So it seems that both basic and non-basic minds are operative in one and the same action. But at first pass, this seems incompatible with REC.” (Weichold 2018, p. 1268).

The problem concerns the presence of two minds and their interaction within the same activity. In Weichold’s analysis, there is a basic mind, concerned with walking, and a content-involving mind, concerned with an explicit intention that is thought to guide the activity of walking. Both minds are co-present in the same act, leading to an “interaction or interface problem”, concerning how the two minds “operative in the same action” are able to forge a connection.

Of course there’s only an interface problem if REC holds, or necessarily leads to, a two-mind picture. But REC, properly understood, does not give rise to such a duality. This is because, for REC, cognition is always and everywhere a matter of organism/environment interaction.^3^ REC understands cognitive activities as “things that we do”, or organism-environment interactions that we are engaged in rather than things that happen in the heads or brains of organisms (as in Noë 2004, see also Myin 2016). As a result, the terms “basic” and “content-involving” qualify activities of organisms or persons. In both cases, these activities are constituted by temporally and spatially extended interactions of an individual with their environment. They are *all* activities and exercises of “abilities”, “competence” and “know-how” (see Sect. 1). Yet activities and abilities can take different shapes in different contexts. In chess contexts, moving an object on a surface can be the activity of developing one’s queen. In a comparable way, abilities to make sounds, in other contexts, can become content-involving abilities.

Basic cognition becomes content-involving in the same way that abilities, know-how and competences develop in general. In a diachronic process of repeated activity, in many but not all cases, one’s competence, when socio-culturally supported and enabled, can grow in both depth and scope. That is, one becomes better adapted to the circumstances and one’s competence can be deployed in a wider array of circumstances.

By construing all cognition as organismic interaction, REC’s outlook is crucially distinct from the picture painted by Weichold. That is, REC does not construe a content-involving activity as one which involves two minds, a basic one and a contentful one “operative in the same activity”. Instead, on REC’s account, there is only one mind, and as a result no synchronic in-the-act connection has to be forged between two components. REC consequently rejects the idea that the mind is ever operative *in* an action let alone that *two* minds are. Rather, REC’s use of the term ‘mind’ is based on the cognitive activities of an organism, basic and content-involving alike. Whether or not some activity of an organism should be seen as broadly mindful, or more narrowly contentful, depends on the way it is diachronically embedded in the rest of the organism’s activities and the normative social practices in which the organism participates. In other words, “content-involving” qualifies an activity-in-a-context.

Compare, for example, a chess player checkmating an opponent and a toddler imitating those same movements without having any understanding of the game. There is obviously an important difference between the player’s and the toddler’s activities, even though superficial descriptions can be alike. For REC, this distinction is not a matter of the presence or absence of chess-related mental representations. Rather, the difference is contextual and historical: the toddler and the chess player interact differently within a different (normative) context and they come to this context via different diachronic paths. A similar analysis pertains to whether content is involved in a particular activity. A movement may involve no content in some contexts and yet be content-involving in other contexts. One can raise one’s arm because of an itch or one can raise it to make the judgment (“Those of you who agree animals have languages of their own, raise your arms”). Importantly, REC does not hold that the difference between the two cases should be analysed in terms of the absence or presence of a contentful component literally *in* the mind or brain of an individual.^4^

Take the example of answering arithmetic questions. Consider someone who makes certain movements to write “5” in response to “3 + 2”. What makes the making of these marks the calculation of a sum, according to REC, is how this activity fits within the wider practice. And what makes it possible for the person to add by marking, continues REC, is that the person has learned, through a gradual and socially embedded process, to adapt her motoric abilities to participation in this social practice. That is, there has been a gradual interweaving of an existing capacity, i.e., the capacity to make marks, with a socially based practice. Therefore, the sensorimotor skills displayed in writing on the blackboard are not independent of the arithmetical skills; instead, the arithmetical skills are structured and sculpted in a history of learning out of these sensorimotor skills (Anderson 2014, p. 232ff). By the time someone masters arithmetic, there is no gap to be bridged between a basic motor mind and a contentful arithmetic mind (which does not mean the skill cannot be improved, or, on the other hand, deteriorate).

Importantly, activities can be shaped by content-involving episodes or activities without *thereby* becoming content-involving. Take the example of a skilled designer who has learned to produce detailed drawings of designs. Learning to make these detailed drawings involves many content-involving activities: it is a skill that is sculpted through explicit instruction and feedback. But this does not mean that the exercise of these drawing skills always involves content. If the skilled drawer absent-mindedly doodles, their drawing activity is not content-involving. But when she makes a sketch in response to somebody asking her what the Eames lounge chair looks like, her drawing activity *can* be evaluated in terms of being correct or incorrect. For example, if she draws a Rietveld chair, she will be mistaken. Thus, whether or not some activity is content-involving depends on the way that activity is situated. The content is consequently not located ‘in’ the drawing. Rather it is a property of the activity and its context, which in this case includes the request to draw the Eames chair.

The question of whether an activity involves content or not can therefore only be answered by considering both the global *and* local context in which the activity takes place. Truth-telling practices are a necessary condition for content to emerge. They enable us to judge that it is true, in most contexts, to say that 2 + 3 is 5 or that this is indeed a drawing of the Eames’ lounge chair. But the background of truth-telling practices is not sufficient for an episode to be content-involving. What is needed in addition is that the activity can be evaluated for semantic correctness. This does not mean the activity must be a judgment. It could be, for example, a question or a non-verbal demonstration (as when one points at a red object to show one understands the concept “red”).

Now we can revisit Weichold’s example of intentionally walking to the theatre. If we follow Weichold and allow that intentionally walking to the theatre should be explained by invoking two components, namely a contentful intention and a contentless motor process, which is in turn guided by the intention, then an interface problem does indeed arise. However, if our ability to act intentionally is instead explained, not in terms of intentions understood as mental things with causal powers, but rather in terms of a history of interactions, such as having learned to speak a language, to obey orders, to make explicit plans and to act according to these plans, then no such interface problem arises.^5^ Consider that there are many activities that a person could engage which would make her walking to the theatre intentional. For example, she could have wondered whether the construction of the theatre was already finished and so decided to have a look, she could have bought a ticket in order to go watch the show, she could have marked the date of the show in her calendar, she could have asked a friend whether she also wants to go to the show, and so on. Any of these earlier activities could be part of the conditions that make her walking to the theatre intentional.

Still, the two-component view Weichold attributes to REC, that is, the view that gives rise to an interface problem, is familiar enough. Indeed, it is often present in the story told to motivate the broadly cognitivist outlook of cognition—an outlook which, as should be clear by now, REC fundamentally opposes. According to a foundational myth of cognitive science, told and retold in countless textbooks (see for example Goldberg and Pessin 2015), there’s a dichotomy between two kinds of psychological processes. The one kind is automatic, rigid, reflex-like and a-contextual, the other flexible, sensitive to context and to cognitive processes and states such as memories, intentions and thoughts. In order to study the former psychological processes, stimulus–response style explanations are suitable. But these stimulus-reponse explanations are thought to have no validity beyond reflex-like behaviour. This is because all psychological phenomena that are not automatic, reflex-like and simple, are informed by intervening representational states, construed along a strong reading of the E-claim, as involving contentful mental representations and contentful information processing (see Nanay 2019 for a recent example of such assumptions at play).

To locate REC’s position in terms of the coordinates of this cognitivist framework is to seriously misconstrue it. In REC’s own framework, there is no occasion for this dichotomy to arise. REC points out that there are no valid reasons to construe basic cognition as “automatic”, “reflex-like” or “simple” at all. Central to REC is the idea that there is cognition that is adaptive, context-sensitive, and flexible *without* involving content. In fact, REC turns the cognitivist view on its head: content does not explain competence; rather, content is rooted in competence The way REC accounts for content is not via the “vertical” overlay of a second “intelligent” layer on top of a first “automatic or reflex-like” layer, but rather via the “horizontal” contextual embedding of activities (Van Dijk and Withagen 2016; Van Dijk and Kiverstein 2020).

REC thus avoids the interface problem à la Weichold by replacing the idea of an “interface” between two things co-present at the same time with the idea of a diachronic integration of capacities, competences and know-how. The problematic assumption underlying the interface problem is what could be dubbed the “matryoshka model” of the development of abilities. According to this model, when a new ability is acquired on the basis of deploying, exercising, refining or extending an existing ability in some new context, the new ability, and any exercise of it, literally contains the old ability and the exercise of it. The more plausible view, and the one REC adheres to, is that the new ability “contains” the old ability only in an analytical sense. In abstracting from the particular context one can discern and point to the old ability. But that doesn’t mean that old ability is a proper, physically distinguishable part of the new ability or that what is added to it so as to achieve a new capacity is literally a physical layer. Doing arithmetic does not contain ‘unformed’ sensorimotor skills that are guided by contents. It instead consists in a historically achieved structuring of sensorimotor skills in a particular context. Moving one’s arm in order to write ‘5’ on the blackboard *is* giving the answer to the arithmetical problem.^6^

At this point, one might object that the view laid out here amounts to a revision rather than an explication of REC.^7^ For how else are we to construe the talk of “basic minds” versus “enculturated, scaffolded minds that are built atop of them” (Hutto and Myin 2013, p. ix)?

Here we must point out that for Hutto and Myin (2013, e.g. p. xviii, p. 14, p. 36, 2017, pp. 90–91, cited in footnote 3), cognition is always a matter of activities. The usage of “mind”, then, is justified by the activities an organism engages in. Content-involving activities are ‘built atop’ basic activities in the sense that they continue from basic activities that are already in place. At the same time, we need different stories if we are to account for these activities: content-involving activities can only be understood with reference to truth-telling practices and involve the question of truth and falsity. This is not the case for basic activities. Still, at no point does REC espouse the view that this means that content-involving activities have to be understood in isolation from basic activities. The two storeys belong to the story, not to the mind.

At the same time, REC has claimed that “introducing content to the cognitive mix (…) does not—*pace* McDowell (1994)—completely overwrite the properties of our basic minds.” (Hutto and Myin 2017, p. 91). Does this not mean that the arrival of content on the scene should be seen as the addition of a contentful layer over an unchanged basic core? It does not. For while it is certainly true that learning to speak, ask questions and make judgments changes how you interact with the world, this does not mean that all cognitive activities, even if they are shaped by the content-involving practices, are themselves content-involving activities. For example, if we are to explain how people dance to disco music, we might have to make reference to content-involving activities–such as writing the movie script for Saturday Night Fever—but that does not mean that disco-dancing itself is a content-involving activity.

## A kink in the cable

REC’s distinction between basic and content-involving cognition has also been criticised for introducing an unacceptable chasm between humans who, according to REC, have content-involving practices and are therefore capable of engaging in contentful activities and other animals that only engage in basic forms of cognition.

A detailed presentation of this critique is found in Moyal-Sharrock (2019). Moyal-Sharrock’s starting point is REC’s claim that the relationship between basic and content-involving cognition is “kinky”. For REC, basic cognition, which is common to humans and other animals, is “different in kind, not merely in degree” (Hutto and Myin 2017, p. 136) from content-involving cognition, which is only found in humans. Therefore, phylogenetically, the development from basic to content-involving cognition, even if it does not contain a gap, contains a kink. REC’s position, as also defended in this paper, is that despite continuity in the processes leading to content-involving cognition, content-involving cognition should be considered as bringing a difference in kind to the cognitive competences of humans and so leads to a functional discontinuity between humans and animals.

Moyal-Sharrock argues that REC has created a fundamental difference which should be avoided. She develops her case in two ways. In some places, she insists that the result of a continuous process cannot be discontinuous with the origins of that process, even if the result and origin are significantly different. In this vein she writes that she initially agrees “with Hutto and Myin that ‘content-involving cognition is a special”, only to add:

As they themselves insist, the sociocultural scaffolding that have enabled the emergence of this higher-level cognition does not imply an inexplicable gap in nature (2017, p. 146), so why attribute kinkiness to an achievement that results from a natural, seamless continuity? (p. 6).

Elsewhere, Moyal-Sharrock resorts to denying that the result—content-involving cognition—is sufficiently different from its origin or from its earlier stages. Animals are not *that* different from humans. They too have social engagements and communication, including symbolic exchanges that are content-involving. A key motivation for Moyal-Sharrock’s claim that human content-involving cognition is nothing special is that language has its roots in this non-linguistic expressive behaviour. She cites Bar-On (2013, p. 39) who claims that expressive animal behaviour acts as a “natural intermediate stage in a diachronic path connecting the completely unminded parts of the animal world with the fully minded, linguistically infused parts that we humans now occupy.”

Moyal-Sharrock also approvingly quotes Wittgenstein: “A child has hurt himself and he cries; and then adults talk to him and teach him exclamations and, later, sentences. They teach the child new pain-behaviour” (Wittgenstein 1953, §244). For Moyal-Sharrock, language is just an extension of such natural expressive behaviour. She points to various forms of animal communication, such as the alarm calls of vervet monkeys, as meaningful, content-involving communication, implicating that it is in no important way different from content-involving human language use. Even the behaviour of crickets, says Moyal-Sharrock (2019, p. 6), shows “that animals are capable of false representation”, because “less desirable smaller males produce courtship calls that dishonestly signal the body size of high condition males in order to be more sexually attractive”.

However, in presenting these two motivations against discontinuity, Moyal-Sharrock does not pause to distinguish between an intuitive notion of “being meaningful” and the notion of “contentful” or “content-involving” as defined by REC. She thereby misses, so we will argue, the reason why REC argues that content-involving cognition is of a different kind from basic cognition. In order to argue against Moyal-Sharrock’s conclusion that REC introduces an unacceptable gap, it is crucial to revisit why REC takes contentful cognition to be of a different kind. For if that reason remains a valid one, then REC’s kinky line remains the right one to walk.

So how does REC’s view of content justify construing content-involving cognition as making a fundamental difference? Here is how we will proceed. We will first explicate what the categorical difference REC proposes comes down to. In doing so, we will focus on the role reflexivity plays in enabling content-involving cognition. We will then turn to why it matters, both philosophically and empirically, to assess the distinction at issue as a fundamental one.

Having content, as per the traditional understanding endorsed by REC, implies having a descriptive, characterizing, or picturing aspect. For some activity R to carry a content about some subject S, R must specify—describe, characterise, or picture S in a way which is evaluable for truth or some related semantic notion.

For example, making the judgment “It is raining in all of Hatfield, Hertfordshire today” answers this requirement: it specifies a specific situation, and what it specifies can be different from what is actually the case. Depending on whether the specified situation relates to the actual situation, the judgement is true or false. Now contrast this judgment with some natural consequence of it raining in Hatfield, Hertfordshire, for example that the soil of Hatfield absorbs drops of water. On all but the most hopelessly crude causal theories of content, this natural consequence merely co-varies with it raining in Hatfield. No content is thereby involved or produced. In short, like the judgement, the consequence is specific to it raining in Hatfield, but, unlike the judgement, it does not by itself *specify* that it is raining in Hatfield, let alone in a truth evaluable way.^8^

So there is a genuine difference between being specific and being specifying. According to REC, this is a crucial distinction (see also Myin 2020). In light of this distinction, we can now reconsider the expressive activities Moyal-Sharrock makes central to her argument, such as the alarm calls of vervet monkeys. Although they are specific, they are not specifying—they don’t involve content. REC provides a straightforward reason for that assessment: these expressive and vocal activities are not activities in truth-telling practices. As a matter of fact, animals, including vervet monkeys, don’t evaluate for truth and falsity or a related semantic notion.^9^ Their behaviour does not involve content because they have no truth-telling practices—just as they can’t produce a checkmate because they have no chess-playing practices, and cannot take out a mortgage because they have no such lending practices.^10^

In contrast, we humans can and do. We can say things, but we can also take an attitude towards saying things. We can, in the words of Jerry Fodor, “have policies” towards our thoughts (or our expressions). As Fodor writes, I can:

make the character of the correspondence between my thoughts and the world *a matter of policy*. Only a creature that is capable of *having policies* with respect to its thoughts can do this sort of thing; in all likelihood, we are the only such creatures (Fodor 1994, 35).

Clark (2013) describes the capability Fodor writes about in the following way:

the capacity (which he thinks is unique to humans) to become aware of the contents of our own thoughts: to not just think that it is raining, but to know that “it is raining” is the content of our thought (p. 180, fn. 4).

Fodor and Clark point to a reflexive relation to the contents of thoughts. Such a relation concerns pre-existing contents that are unveiled by being reflected on. If such pre-existing contents are taken to exist independently from sociocultural practices—as is certainly the case for Fodor—then the notion of content at issue is one REC wants to displace. But, with a twist, reflexivity can still be seen as playing a crucial role in a RECish account of content.

On this account, being able to participate in truth-telling practices requires being able to ask questions such as: ‘Is he correct in saying that?’, ‘How should I use this word?’, and, simply, ‘Is it true to say *x*?’. For content to arise, what is needed are truth-telling practices, understood as reflexive, or meta-linguistic practices, that consist in abilities to talk about talking (see Van den Herik 2019, Ch. 4, for a detailed discussion of this proposal).^11^ Noë (2012, p. 3) explains:

There is no such thing as the naïve, unreflective, theoretically unbiased user of language. For to understand a word is to know how to use it, and that means, among other things, knowing how to explain its use to another, how to answer the question what does this mean? And also how to criticize or correct the usage of others as well as to defend or justify one’s own usage.

In these truth-telling practices, by asking the question ‘Is this true?’, we bind ourselves to standards of correctness that extend beyond our current engagements and depend on the way the world is irrespective of any particular position, perspective, or project. That is, truth-telling practices embody a “familiar intuitive notion of objectivity” (McDowell 1998, p. 222), which means we are ‘beholden to how things actually are’ (Kukla and Lance 2014, p. 22). Note that reference to ‘the way the world is’ does not entail that REC holds that we, as opposed to other animals, somehow have privileged access to a fully pre-determined categorical structure of reality. Rather, it means that we understand, in a practical way, that our judgements can be mistaken, that we can see things in a different light tomorrow, that we can change our mind on the basis of further observation, that others can come to a different judgement, and so on, and so forth.

These reflexive capacities enable us to adopt policies with respect to our own ways of thinking in the Fodorian sense. Being able to adopt these policies, on the REC view, originates in our practices in which these policies are enforced in social interaction. Children are taught not to lie, to question their judgements, to explain the meanings of words, to tell the difference between an elm and a beech, and they are corrected when they mistakenly say that the cat is on the mat when it is in fact sitting beside it. Through these reflexive practices, we instil a sensitivity to the issue of rightness, as Charles Taylor (2016) calls it. Our descriptions can be right or wrong, correct or incorrect, or true or false, depending on the description and the characteristics of the described thing, in a way that goes beyond success or failure in the task at hand. And this policy of trying to get things right is what lies at the heart of our truth-telling practices.

There is a crucial distinction between truth-telling practices and basic communicative practices. As Kukla and Lance (2014, p. 36) explain, animals can engage in highly complex communicative practices that enable them to coordinate their behaviour with respect to their environment, without those practices being truth-telling practices. In these basic communicative practices, the standards of success for the practice are not semantic; Kukla and Lance mention squirrels that hoard nuts. But the same can be said with respect to the vervet monkeys. Their communicative practices in which alarm calls play a role enable them to evade predators and thereby remain alive. As long as they retain their physical integrity, their behaviour is successful. It adds nothing to suppose that the vervet monkey was correct in a semantic sense in responding to this particular event by making this particular alarm call. The only norm of success at play in their practice is that of not becoming prey. The fact that vervet monkeys have different alarm calls that are specific to leopards, eagles, snakes, and baboons (Seyfarth et al. 1980) does not change this fact.

Crucially then, the question of truth, or of being correct, does not arise for the monkeys, and it cannot arise for the monkeys. The reason is that they lack the requisite skills for taking up the reflexive attitude that is required for semantic correctness to get a foothold. And this is where the communicative behaviour of animals is very different from the linguistic^12^ behaviour of humans. For us humans reflecting on the verbal behaviour of ourselves and of others is always a possibility.^13^

One might object that *we* can nevertheless attribute content to the alarm calls of the vervet monkeys.^14^ From the fact that there is a regularity in their communicative behaviour, *we* can say that the monkey incorrectly signalled the presence of a snake in response to seeing a garden hose. But this attributed content does not help us to explain the behaviour of the vervet monkeys, given that the content plays no role *for the monkey*. Here we’d like to say: ‘a wheel that can be turned though nothing else moves with it is not part of the mechanism’ (Wittgenstein 1953, §271). Only when a creature develops a sensitivity to content does it begin to play a role in explaining their behaviour.

It is crucial to keep in mind that, on REC’s position, content is constituted in truth-telling practices. An analogy with other practices can be informative. Take the institution of marriage. We can observe non-human animals engaging in roughly similar behaviour that married humans engage in: they live together, raise children, and so on. If we were to observe this behaviour, we could see this as a precursor perhaps to human practices of marriage. But it does not further our understanding of the non-human animals involved to describe their behaviour in terms of our practices.

REC can thus insist on a categorical difference between content-involving and basic cognition, while still agreeing with Moyal-Sharrock on the idea that it is skills—capacity, competence and know-how—all the way up: that linguistic behaviour is best understood as a further extension of action. Truth-telling practices do not arise in isolation. Plausible theories about the surrounding circumstances are being developed and discussed intensely. They include as crucial elements: already existing forms of communication, an increased need for or advantage of co-operation, processes enhancing social cohesion, norm-abiding and ontogenetic learning in transgenerational settings (see on the philosophical side, Hutto and Satne 2015, on the empirical side, Henrich 2016). For all we currently know, there might be genetic components to the evolution of such circumstances and the capacity to act appropriately in them. Yet there is no need to suppose any of these include the emergence of individual cognitive “superpowers”. Rather, existing capabilities differentiate to become adapted to specific circumstances. So, in that sense, such evolution does not include a major “cognitive leap”—an addition of some novel and unique cognitive mechanism. Nevertheless, the (continuing) evolution of circumstances, and the capacities for operating within them, might lead to something that *is* genuinely functionally discontinuous.

Neither does the process whereby these circumstances and the associated capacities are formed need to have involved individual steps of an extraordinary kind. The reflexive capacities are not magical ingredients. They are simply more skills. Our ability to talk about talking and thereby instil policies towards our own ways of thinking and talking is a form of know-how (Sellars 1971; Van den Herik 2017, 2020). Highlighting the importance of adopting these policies therefore does not introduce a gap into the REC story.

In short, REC can detail what the significant difference between basic and content-involving cognition is. However, perhaps this significant difference should not be considered to be fundamental, or to lead to a difference in kind? We maintain that there are excellent reasons to regard it as a difference in kind. We will highlight two such reasons.

The first is philosophical. REC has always insisted that debates on content and representation can only be productively engaged in on the basis of a well-defined and substantive notion of content. By endorsing a widely accepted notion of content as representation with semantic correctness conditions, REC has criticised representationalist theories of cognition as failing to provide a naturalistically acceptable account of representation. This is because such theories are unable to give a justification why their “representations” deserve this qualification. Indeed, REC accuses them of in fact overintellectualizing: they proceed too fast in labelling natural processes that are merely specific as specifying and thereby representational. This also extends to animal cognition. To claim that nonhuman-animals are capable of contentful behaviour or of false representation, without also making explicit what is the substantive notion of representation at play here, and also why this notion is representational, is to over-intellectualize.

An important motivation, according to REC, to resist over-intellectualization is that it gives rise to fatuous explanations. That is, if truth-conditional content is explained as arising from spuriously ascribed pre-existing truth-conditional content, then nothing is explained at all. In this light, it can be seen that REC’s insistence on the difference between content-involving and basic cognition is compatible with Wittgenstein’s view of language “as rooted in our primitive actions and interactions—not in reason, and not in symbols” and much more so than the view that animals, including insects, engage in contentful behaviour or false representation.

There is a second empirical argument for insisting on the functional discontinuity between content-involving and basic cognition. It consists in pointing to the factual discrepancies between animal cognition and human cognition. One such discrepancy concerns transgenerational learning. Humans but not animals accumulate, refine and expand knowledge over generations. Our content-involving practices make possible forms of cultural evolution that are genuinely and functionally different from anything what exists in the animal kingdom. This difference engenders further differences. We, and not animals, have libraries, calculating machines and the world-wide web. We, and not animals, are able to argue over whether or not evolution is kinky. Content-involving cognition is a prerequisite for these phenomena. These are facts, and they point—quite forcefully—to a positive answer to question of whether the evolution of cognition is kinky. Following our analysis, and resonating with what Fodor, Clark and others have claimed, reflexivity plays a key role in this functional discontinuity. Reflexivity allows us to make the step from basic communication to content-involving talking. It allows us to have a policy towards our sayings and our thoughts. Ultimately, our vast and specific abilities to register, store, manipulate and transfer knowledge have their roots in the capacity to have such policies towards what we say and think. Reflexivity makes content-involving practices relevantly different from other social practices.^15^ In other words, there are good reasons for REC to endorse the idea that a continuous path can lead, via a kink, to a product substantially different from its starting point. Long live the kink!

## Conclusion: same but different

Basic and content-involving activities are the same but different. They are the same because, ultimately, they are both a matter of ability, competence and know-how. Such competences are of organisms and not *in* organisms irrespective of whether content is involved. They don’t split into different components once content arrives on the scene.

Yet even though content-involving cognition is a matter of organismic or personal competence, or of engaging in skilled performance, not all skilled performances are equal. The circumstances determine which cognitive activities are possible. Novel kinds of cognitive capacities can be categorically different from what was available before. Importantly, these novel capacities can give rise to functional discontinuities without there being some inexplicable jump in the processes that gave rise to those discontinuities. Enabled by the context of truth-telling practices, which are themselves dependent on linguistic reflexivity, the capacity to engage in content-involving activities has created a functional discontinuity between animals and humans. Crucially however, this does not cleave the mind in two.

## Footnotes

1 In a similar vein, Wittgenstein elsewhere writes: “ [It] takes remembering to tell us that this is past. But if memory shows us the past, how does it show us that it is the past? It does not show us the past. Any more than our senses show us the present. (1980, 592–593) (for more on Wittgenstein and memory, and the quoted passages, see also Moyal-Sharrock (2009); Myin and Zahidi (2015)).

2 Crucially, the fact that being able to make judgments, once present, changes how one remembers, doesn't imply that from then on, all remembering is content-involving. It isn’t. An episode of remembering can be shaped by one’s content-involving capacities, but not thereby be content-involving. We’ll explicate this further in Sect. 3.

3 Hutto and Myin (2017, p. 91) are very explicit on this: “Although superficially, talk of basic and nonbasic, contentful minds may suggest it, REC denies that there are really two distinct and separate kinds of minds in operation here. By REC’s lights cognition is always dynamic, interactive, and engaging, but in some cases cognitive interactions are also content-involving. Even when cognition involves content and inferential processes the ultimate character of cognition remains enactive and dynamic.”

4 This does not entail that content-involving thoughts cannot figure in explanations according to REC. As described in Sect. 2, REC does not reject the E-claim wholesale but accepts that content-involving activities can be explanantia for other content-involving activities. However, REC conceives of thoughts as episodes of thinking – as activities – rather than as stable states or entities literally within an individual’s mind.

5 This structure will likely be reflected in the bodily and neural make-up of the actor. Nonetheless, these bodily/neural aspects are not to be identified with the intention or with the temporally extended property of the movements “being intentional”.

6 The matryoshka model forms an instance of reification, as does the idea that content is added via the overlay of an extra layer (for recent analyses of such reification, see van Dijk and Withagen (2016) and Zahnoun (2020)).

7 We thank one of our reviewers for insisting on this point.

8 “By itself” should be understood correctly in this context. The judgement *does* specify the situation *by itself*, but that does not mean that it is independent of the broader context. For REC, the ability to judge is explained in terms of the skills obtained by being initiated in certain sociocultural practices, which requires these practices to be there in the first place. This in turn means that, for REC, the content of any particular judgement is constituted by this ontogenetic and phylogenetic history, and cannot be considered in isolation from it.

9 Note that REC’s position is not that, in order to have content-involving practices, one needs to have practices in which the word ‘truth’ plays a role. A similar function can be played, for example, by the English word ‘correct’, when used to semantically evaluate, as in, for example, “It is not correct to say that REC proposes that we have two minds”. This is why REC often talks about correctness conditions in the case of content, which is broader that ‘truth-conditions’. In the case of English, truth conditions are a conspicuous example of the kind of correctness conditions we refer to.

10 Note that REC gives no principled argument against animals having truth-telling practices. In fact, humans *are* animals that have truth-telling practices. However, as far as we know, as a matter of fact, there are no non-human animals who have truth-telling practices.

11 See also T. J. Taylor (2000, 2010) for an account of the central role of reflexivity in linguistic behaviour.

12 Note that REC contends that not all linguistic behaviour is content-involving. The act of greeting someone by saying ‘Hello’, for example, does not specify something in such a way that it can be semantically evaluated.

13 Moyal-Sharrock recognises that something close to what we call reflexivity is crucial for a child to become a competent language-user (yet this does not lead her to agree with REC): “For, grasping a concept is not a matter of connecting a referent to a public symbol, but of acquiring a skill. It is thanks to her acquiring this skill, this normative attitude, that the child is eventually able to go on, on her own; to proceed from other-regulation to self-regulation (Medina 2002, p. 165). Successful enculturation means the child can judge for herself that in a particular instance a word or phrase makes sense, not by comparing it to a benchmark, context-free, use but on the basis of her experience of multiple language-games in which the word or phrase is employed.” (p. 13).

14 We thank an anonymous reviewer for raising this objection.

15 The crucial role of reflexivity in content-involving practices enables us to distinguish them from other normative practices, such as playing chess, so that we are not necessarily committed to the claim that the emergency of any norm-bound practice is kinky.
